# Performance modulation through selective, homogenous surface doping of lanthanum strontium ferrite electrodes revealed by *in situ* PLD impedance measurements†

**DOI:** 10.1039/d1ta08634k

**Published:** 2021-12-03

**Authors:** Christoph Riedl, Matthäus Siebenhofer, Andreas Nenning, Gernot Friedbacher, Maximilian Weiss, Christoph Rameshan, Johannes Bernardi, Andreas Limbeck, Markus Kubicek, Alexander Karl Opitz, Juergen Fleig

**Affiliations:** Institute of Chemical Technologies and Analytics, TU Wien Getreidemarkt 9-E164 1060 Vienna Austria christoph.riedl@tuwien.ac.at alexander.opitz@tuwien.ac.at; Institute of Materials Chemistry, TU Wien Getreidemarkt 9-E165-PC 1060 Vienna Austria; USTEM Universitäre Service-Einrichtung für Transmissions-Elektronenmikroskopie, TU Wien Wiedner Hauptstrasse. 8-10 1040 Wien Austria; CEST Kompetenzzentrum für elektrochemische Oberflächentechnologie GmbH TFZ – Wiener Neustadt Viktor-Kaplan-Strasse 2 2700 Wiener Neustadt Austria

## Abstract

Accelerating the oxygen reduction kinetics of solid oxide fuel cell (SOFC) cathodes is crucial to improve their efficiency and thus to provide the basis for an economically feasible application of intermediate temperature SOFCs. In this work, minor amounts of Pt were doped into lanthanum strontium ferrite (LSF) thin film electrodes to modulate the material's oxygen exchange performance. Surprisingly, Pt was found to be incorporated on the B-site of the perovskite electrode as non metallic Pt^4+^. The polarization resistance of LSF thin film electrodes with and without additional Pt surface doping was compared directly after film growth employing *in situ* electrochemical impedance spectroscopy inside a PLD chamber (*i*-PLD). This technique enables observation of the polarization resistance of pristine electrodes unaltered by degradation or any external contamination of the electrode surface. Moreover, growth of multi-layers of materials with different compositions on the very same single crystalline electrolyte substrate combined with *in situ* impedance measurements allow excellent comparability of different materials. Even a 5 nm layer of Pt doped LSF (1.5 at% Pt), *i.e.* a Pt loading of 80 ng cm^−2^, improved the polarization resistance by a factor of about 2.5. In addition, *p*(O_2_) and temperature dependent impedance measurements on both pure and Pt doped LSF were performed *in situ* and obtained similar activation energies and *p*(O_2_) dependence of the polarization resistance, which allow us to make far reaching mechanistic conclusions indicating that Pt^4+^ introduces additional active sites.

## Abstract 2

The polarization resistance of lanthanum strontium ferrite thin film electrodes with and without additional Pt surface doping was compared directly after film growth by PLD employing *in situ* electrochemical impedance spectroscopy.

## Graphical abstract

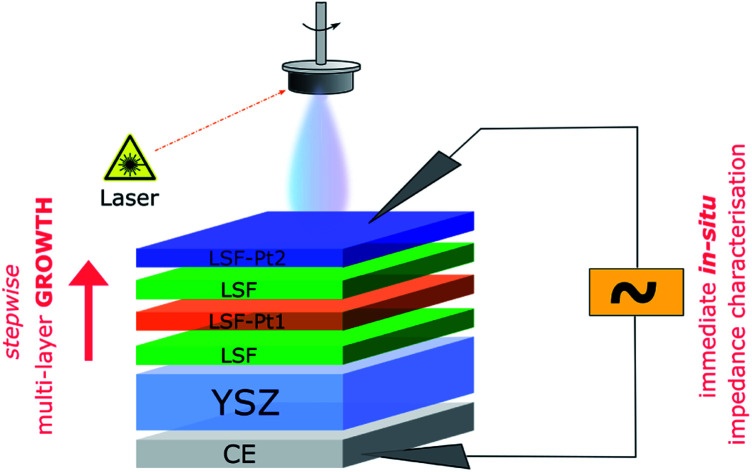

## 1 Introduction

Lowering the operation temperature of solid oxide fuel cells (SOFCs) to intermediate temperatures (400 °C–600 °C) is one of the main goals of current research activities on this topic and requires electrode materials with sufficiently fast surface reaction kinetics even at those relatively low temperatures.^1–6^ While currently La_1−*x*_Sr_*x*_MnO_3−*δ*_ (LSM) or La_1−*x*_Sr_*x*_Co_1−*y*_Fe_*y*_O_3−*δ*_ (LSCF) based composites are used as cathode materials in SOFCs,^7^ recently a study reported exceptionally high power density for SOFCs with La_1−*x*_Sr_*x*_CoO_3−*δ*_ as an air electrode,^8^ which enables a performance increase up to a factor of 10. In the past few years, several studies dealt with either finding new cathode materials or improving the existing ones. Here, mostly perovskite-type mixed ion and electron conducting (MIECs) cathode materials, such as La_1−*x*_Sr_*x*_CoO_3−*δ*_ (LSC),^9–16^ La_1−*x*_Sr_*x*_FeO_3−*δ*_ (LSF),^17–21^ La_1−*x*_Ba_*x*_CoO_3−*δ*_ (LBC),^22,23^ Sm_1−*x*_Sr_*x*_CoO_3−*δ*_ (SSC),^24–26^ La_1−*x*_Sr_*x*_Co_1−*y*_Fe_*y*_O_3−*δ*_ (LSCF),^27–32^ SrTi_1−*x*_Fe_*x*_O_3−*δ*_, (STF)^33–36^ or Ba_1−*x*_Sr_*x*_Co_1−*y*_Fe_*y*_O_3−*δ*_ (BSCF),^37–39^ were investigated. In addition, studies on Pr_1−*x*_Ce_*x*_O_2−*δ*_ (PCO),^40,41^ Nd_2_NiO_4+*δ*_ (NNO)^42,43^ or La_2_NiO_4+*δ*_ (LNO)^44,45^ have been conducted.

While real-life electrodes applied in SOFCs are porous to provide a large electrochemically active surface area, fundamental studies –like this study– are very often carried out on thin film model electrodes grown by pulsed laser deposition (PLD) on single crystalline electrolytes. Studies on porous electrodes mostly aim to optimize the total cell performance, while studies on model electrodes rather try to clarify the underlying reaction mechanisms. Model electrodes further offer the advantages of a well-defined planar geometry, surface area and morphology as well as easy accessibility of the electrode active surface by surface analytical tools.^46^ The simplified and known geometry of such model-type thin film electrodes is key for separating contributions of elementary processes and thus for gaining an in-depth understanding of the reaction kinetics and current pathways on mixed conducting electrode materials. Understanding the elementary processes behind the oxygen exchange reaction (OER) taking place at the surface of MIEC electrodes is crucial for their further improvement. On MIEC electrodes, the elementary steps of the OER (in the cathodic direction) are oxygen gas diffusion, oxygen adsorption on the surface, dissociation and ionization at active sites as well as incorporation of the reduced oxygen species into an oxygen vacancy, followed by ion transport towards the electrolyte.^21,40,46–51^ One important goal of mechanistic OER studies on MIEC electrodes is to identify the rate limiting step and to understand its dependence on the properties of the electrode material and its surface, since this is the basis for a knowledge-driven material improvement. Such knowledge has the potential to greatly contribute to the development of optimized MIEC electrodes for intermediate temperature SOFCs.

Platinum is well known for its favourable redox catalytic properties, which are widely used in a broad range of applications (*e.g.*: oxygen sensors^52–54^ and electrodes in aqueous electrolyte solutions^55,56^). In the context of our study, the usage of platinum or platinum-based catalysts in polymer electrode membrane fuel cell (PEMFC) cathodes to accelerate the oxygen reduction reaction might be most relevant.^57,58^ Lots of efforts have been made to reduce the Pt loading in PEMFCs to about 0.1 mg cm^−2^.^59,60^ Nevertheless, in the field of solid oxide fuel cells, the usage of platinum electrodes is very rare, not only because of costs but also because Pt electrodes generally lead to higher polarisation resistances than mixed conduction oxide electrodes. That might be surprising at first glance, but is related to the fact that in the case of Pt electrodes residing on an oxide ion conducting ceramic, only the region very close to the triple phase boundary (*i.e.* where YSZ electrolyte, Pt and O_2_ meet) is electrochemically active for oxygen reduction, which has been shown in several mechanistic studies.^61–67^

While Pt electrodes on YSZ show rather slow oxygen exchange kinetics, the OER activity of MIEC electrodes can be increased by depositing catalytically active Pt species, which show a high affinity for oxygen splitting and ionization.^68^ In a recent study,^69^ platinum nanoparticles were deposited on the surface of LSF thin film electrodes and a severe decrease of the polarisation resistance was observed, especially at a very low oxyge partial pressure (0.25 mbar O_2_). In addition, a change of the oxygen reduction mechanism was found and the improved oxygen exchange kinetics was explained by a job-splitting with oxygen dissociation taking place on the platinum nanoparticles and oxygen incorporation occurring at the free surface of LSF. While metallic nanoparticles sputter deposited on a MIEC surface are usually relatively large, with diameters of more than ten nanometers,^69^ incorporation of the catalytically active platinum group elements into the lattice of the pervoskite-type MIEC (*i.e.* doping) may offer the advantage of achieving exsolved nanoclusters or even single atoms of the catalyst dopants in the electrode material. With a high dispersion of the metal, the catalytically advantageous properties of platinum may then be most efficiently utilised.

Several studies have already shown that the performance of established SOFC electrodes can be improved by doping with additional catalytically active elements. For example, studies on SrTi_*x*_Fe_1−*x*_O_3−*δ*_ revealed positive effects of Co,^70^ Ni^71,72^ or Ru^73^ doping on the polarisation resistance of the electrode and showed increased long term stability. Similar effects were found on La_1−*x*_Sr_*x*_CrO_3−*δ*_ after doping with Ru or Ni.^74^ In addition, “self-regenerating” automotive catalysts were developed by doping a Pt, Rd or Pd metal into the perovskite host lattice. While highly active exsolved nanoparticles were found under reducing conditions on the catalyst surface, studies showed that these nanoparticles were reincorporated into the host lattice under oxidizing conditions.^75–77^

In this study, we reveal how minor amounts of platinum surface doping (of the order of only 1% of the overall cation amount) can strongly decrease the polarization resistance of lanthanum strontium ferrite (LSF) thin film electrodes grown by pulsed laser deposition (PLD). Moreover, the fabrication of multilayer electrodes allowed us to directly compare the electro-catalytic activity of electrodes with different surface compositions (in this study, pure LSF electrodes and Pt doped LSF electrodes). For electrochemical characterization, impedance measurements were conducted *in situ* on thin film electrodes directly inside the setup of the PLD immediately after growth (*i*-PLD). This technique was already employed successfully in several studies^78–82^ and enables characterizing electrodes unaltered by externally triggered degradation effects. The unusually high stability of the polarisation resistance in *i*-PLD studies enables its observation over a longer period of time and allowed us to study the electrode kinetics at different *p*(O_2_) and temperatures, thus leading to additional mechanistic insights into the OER on the surface of LSF with and without Pt. In order to establish correlations between the surface composition and the electrocatalytic activity of the electrode, *in situ* ambient pressure X-ray photoelectron spectroscopy (AP-XPS) was performed under operation conditions. In addition, inductively coupled plasma mass spectroscopy (ICP-MS) was used to determine the total platinum amount in the thin films. Atomic force microscopy (AFM) and transmission electron microscopy (TEM) were used to reveal the surface and bulk morphology of the electrodes and the obtained results support our mechanistic interpretations.

## 2 Experimental

### 2.1 Sample preparation

LSF and Pt-doped LSF powders were prepared *via* the Pechini route^83^ from Fe, La_2_O_3_, SrCO_3_ and Pt(NO_3_)_2_(NH_3_)_4_ (all bought from Sigma Aldrich, at least 99.99,% purity). For the doped material, two different Pt concentrations – nominally 0.9 at% and 1.5 at% of cations – were chosen, which are denoted LSF-Pt1 and LSF-Pt2, respectively. The obtained powders were calcined at 800 °C in air, pressed into PLD targets by cold-isostatic pressing at 150 MPa and sintered at 1200 °C in air for 12 h. As electrolyte substrates, (100)-oriented yttria stabilized zirconia single crystals (YSZ) (0.5 × 0.5 × 0.05 cm^3^) (CrysTec, Germany) for polycrystalline film growth and (100)-oriented La_0.95_Sr_0.05_Ga_0.95_Mg_0.05_O_3−*δ*_ (LSGM) single crystals for epitaxial film growth were used.^84^ Before deposition of oxide thin films, current collecting platinum grids (with 15∣5 μm or 25∣10 μm mesh∣strip width) were prepared on the single crystals by lift-off lithography and subsequent magnetron sputtering of 5 nm titanium (as an adhesion promoter) and 100 nm platinum (BAL-TEC MED 020, Liechtenstein). As a counter electrode, nano-porous La_0.6_Sr_0.4_CoO_3−*δ*_ was deposited by PLD at 450 °C and 0.4 mbar O_2_ with a target to substrate distance of 5 cm. This leads to a kinetically very fast electrode with almost negligible polarisation resistance compared to a dense working electrode. The working electrodes (*i.e.* LSF, LSF-Pt1, and LSF-Pt2) were grown on the single crystal substrates with current collector grids by ablating the respective targets with a KrF excimer laser (Compex Pro 201F, wavelength 248 nm). Multilayers of LSF, LSF-Pt1 and LSF-Pt2 were grown during the *i*-PLD characterisation. The substrate temperature (measured using a pyrometer) and oxygen background pressure were 600 °C and 0.04 mbar O_2_, respectively. A film thickness of 10 nm was obtained after applying 1000 pulses with a laser frequency of 2 Hz, a laser fluence of 1.1 J cm^−2^ (measured inside the PLD chamber), and a target to substrate distance of 6 cm. The growth rate was determined using measurements obtained with a profilometer (DekTakXT, Bruker, USA) after the deposition of a known number of pulses.

### 2.2 Electrochemical measurements

**Fig. 1:**
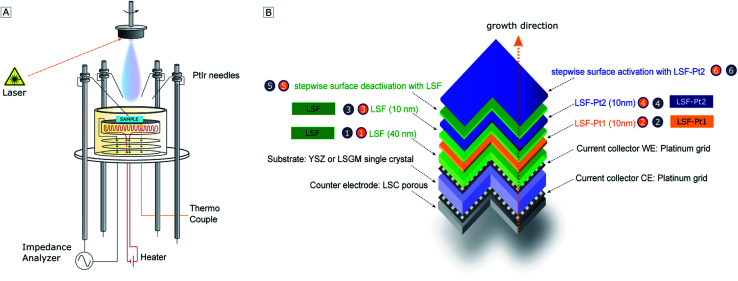
(A) Sketch of the *i*-PLD heater. (B) Sketch of the multi-layer structure of the *i*-PLD sample grown on YSZ or LSGM single electrodes. This nomenclature is identical in Fig. 1, 6 and 7 and the numbers are also mentioned in the text.

*In situ* electrochemical measurements during PLD deposition (*i*-PLD) were conducted with an Alpha-A High Performance Frequency Analyzer equipped with an Electrochemical Test Station POT/GAL 30V/2A setup (both Novocontrol Technologies, Germany). For *i*-PLD measurements, a sample was placed on the PLD heater and covered with a corundum mask (cutout 0.45 × 0.45 cm^2^) to prevent short circuiting of the working electrode and counter electrode *via* the edges of the sample during the subsequent film deposition. The working electrode was brought into contact with a PtIr-needle and the counter electrode *via* platinum paste brushed onto the heater. A sketch of the measurement setup can be found in Fig. 1.

Impedance measurements were typically conducted in a frequency range from 10^6^ to 10^−1^ Hz (if needed for resolving the electrode feature, the frequency range was extended to 10^−2^ Hz), with an AC amplitude of 10 mV root-mean-square, and a resolution of 5 or 10 points per frequency decade. In many cases, oxygen partial pressure and temperature were kept at 600 °C and 0.04 mbar O_2_ for deposition; in some cases pressure and temperature were varied for a more detailed impedance characterisation.

### 2.3 Characterisation of thin film surface and bulk composition, structure, and morphology

X-ray photoelectron spectra (XPS) were acquired using a lab-based AP-XPS machine^85^ with monochromated Al k-alpha radiation. For better comparability of the different Pt oxidation states, the spectra were corrected for the Fermi level shift of the grounded working electrode that is introduced by electrochemical polarization, or atmospheric variations.^86,87^ Due to the absence of carbon, the binding energy was calibrated to the main O 1s component at 529.8 eV. Quantification was performed using Scofield cross-sections^88^ and S-type “Shirley” backgrounds with the software “Casa XPS”. In order to achieve an equal probing depth for all elements, we compared peaks with similar photoelectron energy (and an effective attenuation length of *ca.* 1.8 nm), Sr 3d (BE 130 eV), La 4d (BE 110 eV), Pt 4f (BE 75 eV) and Fe 3p (BE 58 eV).

Two modes were used for analysing the surface chemistry of LSF-Pt2 electrodes: the XPS machine was operated either in UHV or the sample was exposed to 1 mbar O_2_ (AP-XPS mode). In the latter case, the sample was heated to a typical operation temperature (600 °C) and a partly even bias voltage was applied (leaving an overpotential of ±300 mV) in order to drive the electrode into the oxygen reduction or oxygen evolution mode.

Atomic force microscopy (AFM) was performed in tapping mode with a Nanoscope V multimode setup (Bruker, United States of America) over a scan area of 1 × 1 μm^2^. Data were plotted with python™.

For measuring the bulk composition of the deposited films, 100 nm thin layers were prepared on YSZ substrates (without a Pt grid and a counter electrode). The samples were then digested in triplicates with 0.5 mL aqua regia (HCl : HNO_3_ = 3 : 1) by heating to 60 °C for half an hour in falcon tubes and then diluted to a volume of 10 mL with millipure water. All chemicals were purchased from Merck in the highest available purity, and single element ICP standards were also purchased from Merck. For the analytical experiments, samples were diluted 1 : 100 with 1 v% HNO_3_ with 1 ng g^−1^ indium as the internal standard. The internal standard was used to correct instrumental drift during the course of the measurement. Measurements were conducted on an iCAP-Q ICP-MS instrument (Thermo Scientific, United States of America) with an ESI SC-2-DX autosampler (ESI, USA) and a Fast valve (ESI, USA), see ref. 89 for detailed instrument parameters. The following ions were monitored during the measurement: ^57^Fe^+^, ^139^La^+^, ^86^Sr^+^, ^88^Sr^+^, ^194^Pt^+^, ^196^Pt^+^ and ^115^In^+^. Matrix matched external standards were used for quantification of background corrected signals.

Scanning electron microscopy (SEM) measurements were carried out with a FEI Quanta 250 FEGSEM. A thin electron transparent lamella was produced using a ThermoFisher Scios 2 Dual Beam FIB. In order to protect the surface of the lamella, a thin C-layer (about 35 nm) was deposited first by electron beam deposition followed by a W-protection layer. For transmission electron microscopy (TEM), a TECNAI F20 field emission TEM was used equipped with a high angle annular dark field detector (HAADF) in scanning transmission (STEM) mode and a Rio 16 CMOS Camera in TEM mode, respectively.

Grazing incidence X-ray diffraction (XRD) measurements were performed with an Empyrean X-ray diffractometer (Malvern Panalytical, GB) equipped with a parallel beam mirror on the incident beam side and a parallel plate collimator and a scintillation detector on the diffracted beam side.

## 3 Results

### 3.1 Stoichiometric composition of the electrodes

The compositions of the PLD-grown LSF and LSF-Pt thin films were determined by acidic digestion of the entire films and quantification of the cations in the resulting solution using ICP-MS. A thin film stoichiometry of La_0.5207±0.008_Sr_0.4793±0.008_Fe_1.0_O_3−*δ*_ for LSF, La_0.5285±0.004_Sr_0.4715±0.004_Fe_0.9817±0.001_Pt_0.0183±0.001_O_3−*δ*_ for LSF-Pt1 and La_0.5207±0.008_Sr_0.4747±0.004_Fe_0.9728±0.001_Pt_0.0272±0.001_O_3−*δ*_ for LSF-Pt2 was found. Thus from ICP-MS measurements, we assumed about 0.9 at% of all cations of LSF-Pt1 and 1.4 at% of LSF-Pt2 to be platinum.

In order to verify these results, X-ray photoelectron spectroscopy (XPS) measurements were carried out in an UHV on pristine thin films directly after PLD deposition. From the XPS spectra, 0.9 at% and 1.5 at% of the total cation amount of LSF-Pt1 and LSF-Pt2 were identified to be platinum, respectively. This very good agreement of the surface sensitive XPS results with bulk composition suggests the absence of any noteworthy surface accumulation or depletion of Pt. The binding energies (77.5 eV and 74.0 eV) found for Pt-4f_5/2_ and Pt-4f_7/2_ were in good accordance with the literature data^90^ for Pt^4+^. Accordingly, we conclude that platinum was successfully incorporated into the perovskite lattice of LSF. This is also in accordance with the results of several other characterisation techniques, which did not yield an indication for a possible phase separation (for details, see below). Assuming B-site occupation of Pt^4+^, we thus have about 2% and 3% of Pt on this site in the two sample types (LSF-Pt1 and LSF-Pt2).

### 3.2 Structural and morphological characterization of the electrodes

**Fig. 2:**
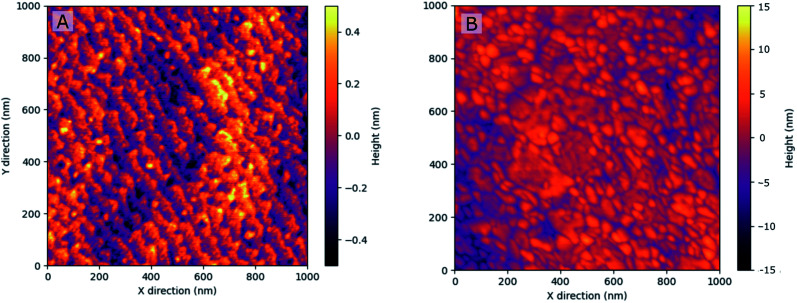
AFM image of the measured multilayer electrodes grown on LSGM (A) and YSZ (B) with a top layer made of LSF-Pt2 (10 nm). Please note the different scale bars.

For surface morphology characterization, atomic force microscopy (AFM) measurements were conducted on multi-layer electrodes grown on La_0.95_Sr_0.05_Ga_0.95_Mg_0.05_O_3−*δ*_ (LSGM) and yttria stabilized zirconia (YSZ) substrates. As depicted in Fig. 2, epitaxial film growth with clearly visible atomic steps (height: ∼0.4 nm = 1 lattice constant) was observed on LSGM, while polycrystalline film growth was found on YSZ. On neither substrate the deposited thin films show any indication for phase separation such as occurrence of surface particles, pinholes or cracks (please see the ESI† for SEM and additional AFM images of the pristine and degraded electrodes (Fig. S5†)).

**Fig. 3:**
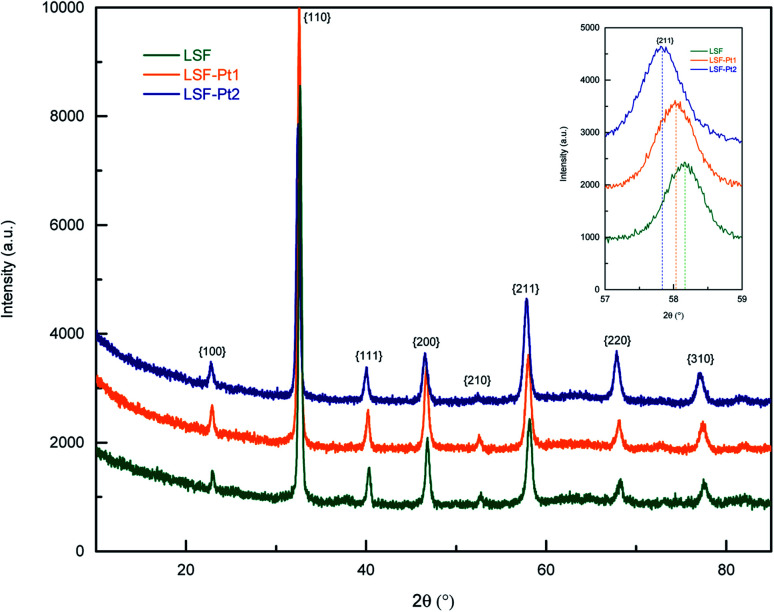
Diffractogram of pristine LSF (green), LSF-Pt1 (orange) and LSF-Pt2 (blue) thin films deposited on YSZ single crystals. The inset shows a magnification of the 211 reflex.

The polycrystalline growth of the thin films on YSZ was also revealed by XRD measurements in gracing incidence mode. As visible in Fig. 3, especially at higher 2*θ* values a small shift of the reflexes to lower 2*θ* was observed. This reveals that incorporation of platinum leads to a slight enlargement of the LSF unit cell.

**Fig. 4:**
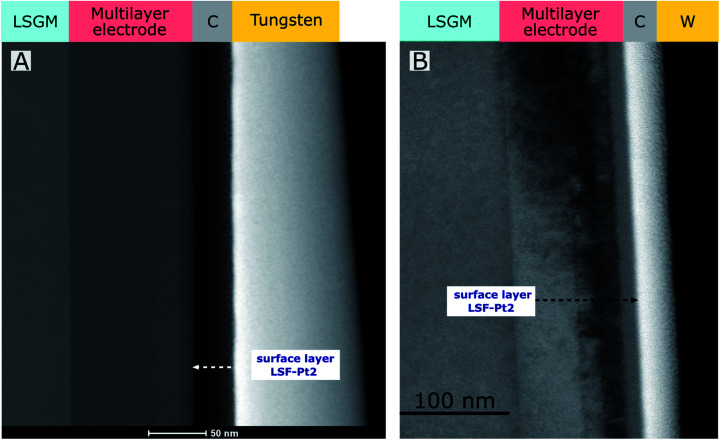
(A) HAADF-STEM and (B) TEM image of a multilayer electrode (top layer: LSF-Pt2). No exsolutions of platinum nanoparticles are visible on the electrode surface.

Transmission electron microscopy (TEM) was conducted to study the bulk and surface morphology of the electrode. In Fig. 4, a HAADF-STEM image (A) and a TEM image (B) of a multilayer electrode grown on LSGM consisting of several layers of LSF, LSF-Pt1 and LSF-Pt2 with a top layer made from LSF-Pt2 can be found. The structure of the electrode is sketched in Fig. 1 and the electrochemical performance of the different layers is shown in Fig. 7. In accordance with AFM and SEM measurements, also HAADF-STEM and TEM measurements confirmed that platinum is well incorporated into the perovskite host lattice and significant formation of platinum exsolution on the electrode surface can be excluded. The contrast in HAADF-STEM is related to the thickness, (average) atomic number and density of the sample position. In Fig. 4(A) the individual layers in the multi-layer electrode appear uniform. No formation of Pt nanoparticles is visible, which would be seen as brighter regions.

### 3.3 Ambient pressure XPS measurements

**Fig. 5:**
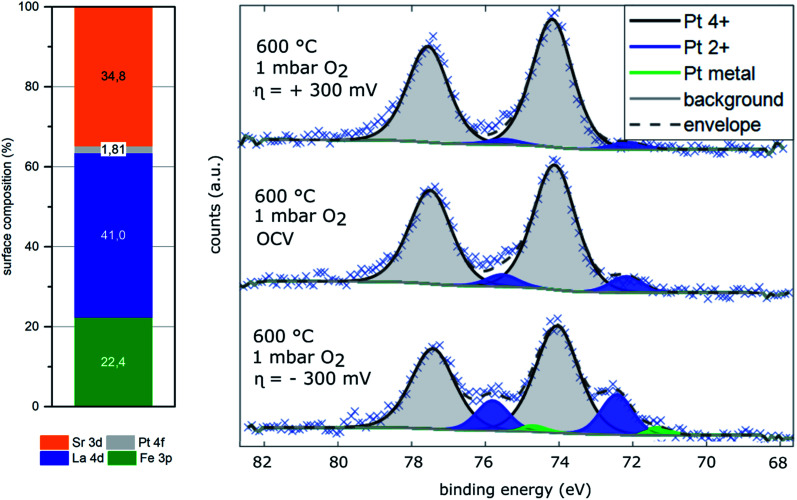
Results of ambient pressure XPS (AP-XPS) measurements on a LSF-Pt2 electrode under open circuit voltage and with polarization. Observed binding energies of platinum and distribution of the platinum oxidation states under different working electrode overpotentials *η*.

In addition to XPS characterization in UHV, ambient pressure XPS (AP-XPS) measurements were conducted at 600 °C in 1 mbar O_2_ to study the surface composition of the LSF-Pt2 electrode under conditions very similar to the *i*-PLD impedance measurements. In good accordance with *ex situ* XPS measurements conducted on LSF-Pt2 in UHV, where 1.5 at% of the surface composition was found to be platinum, AP-XPS detected 1.8 at% of the cations in the near surface region to be platinum. AP-XPS was further used to study the oxidation states of platinum under operation conditions. As shown in Fig. 5, also AP-XPS confirmed that under oxidizing conditions platinum is present in the oxidized form as Pt^4+^ (97%) and Pt^2+^ (3%), thus further supporting the interpretation the noble metal being incorporated into the perovskite-type oxide and, most probably, occupying the B site. Since device limitations do not allow oxygen partial pressures higher than 1 mbar O_2_ for AP-XPS measurements, an electrochemical overpotential was applied to systematically modify the point defect concentration of the thin film working electrodes and thus to achieve higher as well as lower effective oxygen partial pressures in the electrodes. For a surface limited working electrode (WE) and a reversible counter electrode (CE) the effective oxygen partial pressure in the WE (*p*O_2_^WE^) depends on the WE overpotential *η* as given in eqn (1). This requires the absence of significant transport limitations and of charge transfer polarisation at the electrolyte interface, which is fulfilled in our films. For a detailed explanation, please see ref. 86, 91 and 92.1
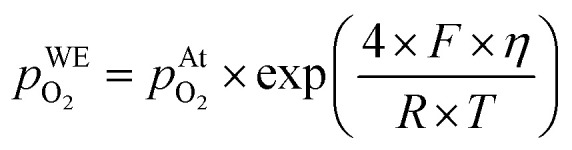


where *p*O_2_^At^ is the oxygen partial pressure of the surrounding atmosphere in the single chamber setup, *F* is Faraday's constant, *R* is the gas constant, and *T* is the absolute temperature. The working electrode's overpotential was obtained by subtracting the ohmic voltage drop (electrolyte) from the applied voltage.

2 *η* = *U*_set_ − *I* × *R*_ohm_

An anodic overpotential of 300 mV at 1 mbar O_2_, which is equivalent to about 8 orders of magnitude higher effective *p*(O_2_) did not lead to any significant changes of the oxidation states of platinum. On the other hand, the application of a −300 mV cathodic overpotential (about 8 orders of magnitude lower effective *p*(O_2_)) clearly revealed changes in the platinum oxidation states. As visible in Fig. 5, platinum metal (3%) and Pt^2+^ (20%) together with Pt^4+^ (77%) were found on the cathodically polarized surface of the electrode. This may be an indication for the onset of an exsolution process of metallic Pt under these reducing conditions.

It is further worth mentioning that our surface chemistry and surface morphology results obtained by AFM, SEM, TEM and AP-XPS measurements on Pt containing LSF are in excellent accordance with the literature.^75–77,93^ Different studies on noble metal doped pervoskites also showed that noble metals can be incorporated into the pervoskite lattice under oxidizing conditions. Only under reducing conditions, which are not topic of this study, metallic nanoparticles were found on the surface of the materials.

### 3.4 *In situ* electrochemical characterisation during growth of multi-layers

Multi-layer structures of LSF, LSF-Pt1 and LSF-Pt2 were sequentially deposited on single crystals of YSZ (for polycrystalline film growth) and LSGM (for epitaxial film growth). The polarization resistance of the obtained electrodes was continuously characterized by impedance spectroscopy in the PLD chamber, *i.e.* instantly after each film growth at deposition pressure and temperature. The required *i*-PLD setup, which was used for sample heating and electronic contacting of the working electrode with a PtIr needle, is shown in Fig. 1A. Our measurements show excellent reproducibility, as for each substrate all the thin films were deposited at the exactly same temperature (600 °C), the same oxygen partial pressure (0.04 mbar O_2_) and without any external influences (such as humidity, CO_2_, or impurities from ambient air) and many reasons for a scattering of polarization resistance are avoided.

**Fig. 6:**
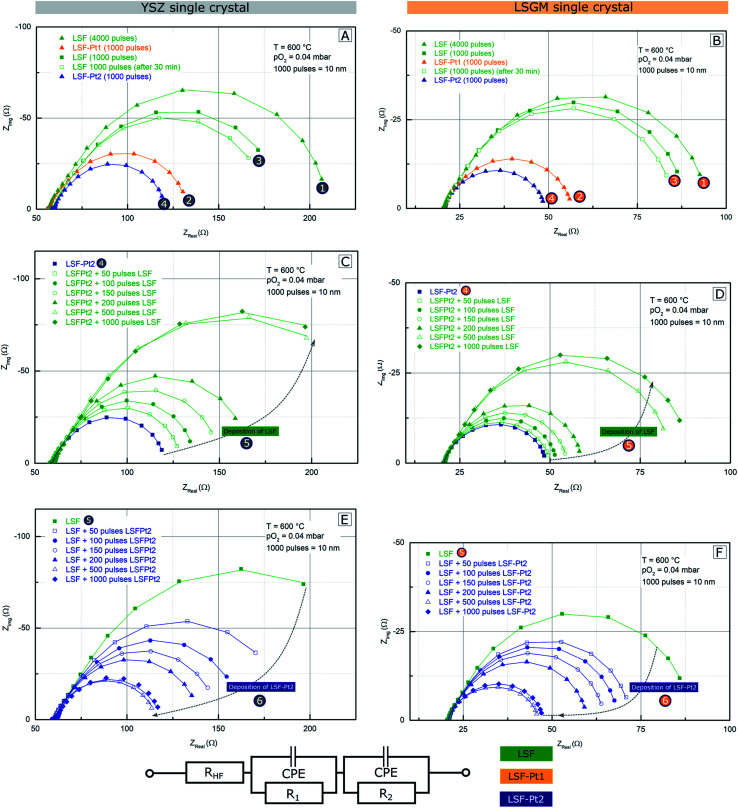
Measured impedance spectra from an *i*-PLD multi-layer experiment grown on YSZ (A, C and E) and LSGM (B, D and F) single crystals. Symbols are the measured data, lines are fits to the equivalent circuit shown. (A) and (B) Initial deposition of the LSF, LSF-Pt1, and LSF-Pt2 electrodes (10 nm each). (C) and (D) Stepwise deactivation of a LSF-Pt2 electrode by depositing LSF on its surface. (E) and (F) Stepwise surface activation of a LSF electrode by growing LSF-Pt2 on top. The obtained area specific resistances of the electrode surface are summarized in Fig. 7. The encircled numbers refer to certain steps in the course of the *i*-PLD experiment and details of the individual steps are explained in the text.

**Fig. 7:**
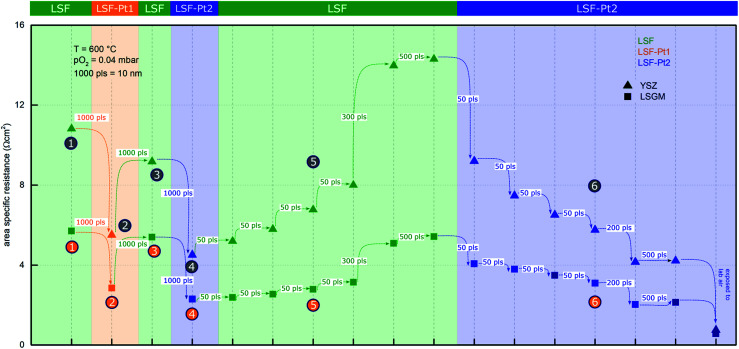
Area specific resistance of *i*-PLD multi-layer electrodes grown on YSZ (triangles, top row) and LSGM (squares, bottom row) calculated from the spectra shown in Fig. 6. Green symbols and lines display deposition of LSF, blue symbols and lines display deposition of LSF-Pt2, orange symbols and lines display deposition of LSF-Pt1.

In this section, *i*-PLD experiments on LSF/Pt-LSF multi-layers are shown, which demonstrate that extremely small amounts of Pt surface doping can already strongly improve the oxygen exchange kinetics. The main reason for preparing multi-layer structures in combination with *i*-PLD measurements is the direct comparison of the polarization resistances of different LSF electrodes surfaces (pure and Pt doped) on the very same substrate. The structure of a deposited multi-layer electrode is sketched in Fig. 1B. The impedance spectra measured upon growth of such a multilayered electrode are displayed in Fig. 6. In order to improve the readability of the impedance results, all data in Fig. 6 and 7 dealing with LSF are plotted in green, while the data of LSF-Pt1 and LSF-Pt2 are plotted in orange and blue, respectively. All spectra exhibit a high-frequency intercept, which can be assigned to the transport of O^2−^ ions through the single crystalline YSZ or LSGM electrolyte substrate.^84,94^ Due to different oxygen ion conductivities of the two substrates, different high frequency resistance values were found on YSZ (53 Ω) and LSGM (23 Ω). These values are in excellent accordance with the conductivity of YSZ^94^ and LSGM^84^ at 600 °C reported in the literature.

According to many literature studies on similar materials (LSF, LSC, and LSCF), the main arc in the impedance spectra is related to the oxygen exchange kinetics on the electrode surface,^17,24,33,40^ as long as electronic in-plane conductivity and ionic across-plane conductivity are sufficiently high, which is the case for our films. In the medium frequency range, sometimes a shoulder was observed, which is most likely associated with the ion transfer across the electrode/electrolyte interface.^24^ The formation of detrimental amounts of SrZrO_3_ between the electrode and the YSZ single crystal was excluded in accordance with earlier studies at 600 °C (ref. 17, 69 and 79) and measurements indicating the formation of SrZrO_3_ at temperatures above 800 °C.^95^ The impedance spectra were fitted to the equivalent circuit shown in Fig. 6, where one of the *R*|CPE elements (CPE = constant phase element^96^) represents the low frequency surface feature of the electrode impedance, and the other one the medium frequency interface shoulder. Although this circuit only approximates the mechanistic model,^24^ we chose this more robust circuit for the sake of obtaining a stable fit, since the interface feature was not well established for all the measurements and especially the more interesting low frequency arc can thus be fitted with high accuracy. The most interesting fit parameter extracted from the low frequency feature is the surface polarization resistance, which characterizes the activity of the electrode surface for oxygen exchange. For better comparability, it was referred to the active electrode area (thus yielding an area specific surface resistance, “surface ASR”), as prior studies have shown that only the area above the YSZ is active for the oxygen exchange reaction, while the area above the platinum grid is inactive due to a relative large in-plane ionic resistance in the LSF film^17^ (images of the platinum grid structure can be found in the ESI, Fig. S8†). The resulting surface ASR values for the entire course of the *i*-PLD experiment are summarized in Fig. 7 for both electrolytes and for all terminating surface layers achieved by *in situ* deposition of pure LSF and Pt doped LSF.

Let us first focus on the results obtained on YSZ substrates (see the left column of Fig. 6). In the beginning of the shown *i*-PLD experiment 4000 pulses (= 40 nm) LSF were deposited on YSZ and the impedance of the electrode was measured immediately at 600 °C and 0.04 mbar O_2_ – see number ① in Fig. 6A and B. Please note that encircled numbers are used in Fig. 1, 6 and 7 and throughout the text to identify each subsection of the *i*-PLD experiment. The subsequent deposition of 1000 pulses (= 10 nm) LSF-Pt1 on the LSF electrode caused a decrease of the surface-ASR from 10.8 to 5.7 Ω cm^2^; see ②. The electrode was then “deactivated” to 9.2 Ω cm^2^ by depositing 1000 pulses of LSF; see ③. As visible in Fig. 6, the polarisation resistance slightly decreased after an annealing time of 30 min and saturated at 9 Ω cm^2^. This might be explained by Pt diffusing through the thin LSF top layer (10 nm) to the surface of the electrode. However, this effect is small compared to the performance boost achieved by the general effect of Pt doping and was thus not further investigated.

In the next step, the electrode was reactivated again by 1000 pulses of LSF-Pt2 to 4.5 Ω cm^2^; see ④.

LSF was stepwise deposited in small amounts (50, 50, 50, 50, 300, and 500 pulses) on the terminating LSF-Pt2 layer of the already existing heterostructure and accordingly a stepwise increase of the polarization resistance was observed – see number ⑤ in Fig. 6C. After a total of 500 pulses, a polarisation resistance of 14.1 Ω cm^2^ was found, which just slightly increased to 14.3 Ω cm^2^ after depositing another 500 pulses of LSF. This shows that a 5 nm thick surface layer is sufficient to imprint the thin film electrode with the surface kinetics of LSF.

*Vice versa* the electrode performance was subsequently again improved by another stepwise deposition of LSF-Pt2 until a polarization resistance of 4.2 Ω cm^2^ was reached; see ⑥ in Fig. 6E. Remarkably, already the deposition of the first 50–100 pulses LSF-Pt2, which corresponds to only 0.5 nm–1 nm of the deposited material, led to a significant drop of the polarization resistance. This result points out the outstanding effectiveness of surface doping, which may point to future pathways of highly efficient precious metal utilization also in solid oxide cells. Again, after a total of 500 pulses of LSF-Pt2 deposition, the polarization resistance of the electrode reached a virtually constant value and no further significant changes could be observed after deposition of another 500 pulses of LSF-Pt2. This is highly remarkable because it indicates that already *ca.* 1.5 at% Pt cations (= 3% Pt on B site) within 5 nm can more than double the reaction rate compared to pure LSF. The Pt amount corresponds to 80 ng cm^−2^, which is drastically lower compared to a typical Pt loading in PEM fuel cells, with is often in the range of 0.1 mg cm^−2^.^60^

The entire *i*-PLD multilayer experiment was repeated in exactly the same way with LSGM as the electrolyte. The measurements revealed that LSF with epitaxial structure shows superior oxygen reduction kinetics (5.7 Ω cm^2^ compared to 10.8 Ω cm^2^ for LSF in the polycrystalline structure). However, as evident from the impedance spectra in Fig. 6B, D, and F the qualitative behavior of the electrodes is identical. Roughly speaking the surface modification with LSF-Pt again improves the performance by about a factor of 2–2.5 (2.8 Ω cm^2^ for LSF-Pt1 and 2.3 Ω cm^2^ for LSF-Pt2). The difference between films on YSZ and on LSGM is attributed to the fact that LSF on YSZ grows polycrystals, while LSGM supports epitaxial film growth. The observed surface ASR difference is also in accordance with the literature, where epitaxially strained thin films showed different oxygen exchange kinetics than unstrained, polycrystalline thin films. While tensile strain is known to promote oxygen reduction, compressive strain was found to decrease oxygen exchange activities.^42,81,97^

Moreover, the long-term stability of LSF-Pt2 in the setup of the *i*-PLD electrodes was investigated and compared to LSF. After annealing LSF-Pt2 electrodes for 16 h in synthetic air at 600 °C, the formation of particles on the electrode surface was observed. In addition, XPS measurements revealed a strong increase of the platinum surface composition after annealing together with a shift of the binding energy, which indicates the observed particles to be metallic Pt. Additional *i*-PLD measurements show that as Pt diffuses to the surface, also the beneficial effect of Pt doping is lost and over the time an increase of the surface-ASR is found. However, since in this study *i*-PLD measurements allowed us to characterize pristine electrodes directly after PLD deposition, the observed low long term stability does not affect the mechanistic conclusions made in this study. For a more detailed description of this Pt related degradation, please refer to the ESI, Chapter 1.†

### 3.5 *p*(O_2_) and temperature dependence of LSF and LSF-Pt2 polarisation resistance

**Fig. 8:**
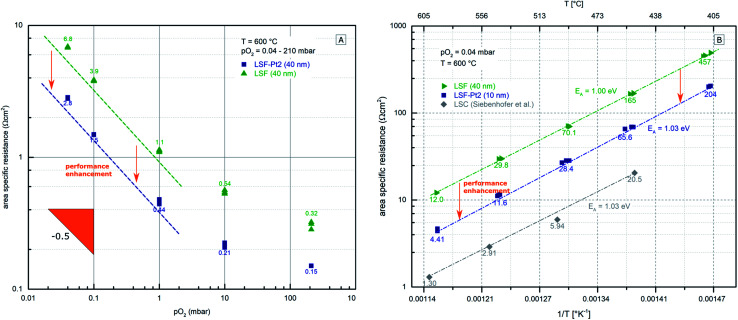
(A) Surface-ASR of LSF (green) and LSF-Pt2 (blue) (two different samples) *versus* the oxygen partial pressure plotted as log/log diagram. The numbers next to the symbols are the mean ASR values in Ω cm^2^. Lines are a guide for the eye. (B) Area specific resistance plotted *versus* inverse temperature for LSF (green), LSF-Pt2 deposited on top of LSF (blue) and LSC (grey). Data for LSC are taken from ref. 79 for comparison.

Owing to the high reproducibility and sufficient stability of the electrode kinetics in the *i*-PLD experiments (see the ESI, Fig. S1†), very reliable *p*(O_2_) and *T* dependencies of the oxygen exchange reaction can also be obtained. Those are highly interesting, since they offer the possibility of improving the mechanistic understanding of the oxygen reduction kinetics on pervoskite-type electrodes. Hence, the dependence of the surface ASR on the oxygen partial pressure was studied in the *i*-PLD setup on 40 nm thick LSF and LSF-Pt2 electrodes freshly deposited on two different YSZ substrates. YSZ substrates were chosen for the reason of better comparability with the literature data, which are primarily available for this type of electrolyte. Impedance measurements were carried out at 0.04 mbar, 0.1 mbar, 1 mbar, 10 mbar, and 210 mbar O_2_ by adjusting the oxygen flow in the PLD chamber at 600 °C. The obtained surface-ASR values are plotted on a log/log scale *versus* p(O_2_) in Fig. 8A. In accordance with measurements from the multi-layer experiment (see Fig. 7), LSF-Pt2 showed around a factor of 2.5 faster oxygen reduction kinetics than pure LSF. Surprisingly, LSF and LSF-Pt2 exhibit the very same oxygen partial pressure dependence, as visible in Fig. 8A. However, the *p*O_2_ dependence cannot be described by a single slope in this *p*O_2_ range. Rather, the line strongly flattens towards higher *p*O_2_, with a slope >0.5 at low pressures and little dependence at high *p*O_2_.

In addition, the temperature dependence of the surface ASR was investigated on both LSF and LSF-Pt2. To do so, temperature dependent impedance measurements were first conducted on a 40 nm LSF electrode. Subsequently, 10 nm of LSF-Pt2 was deposited onto the electrode and the temperature dependent measurements were repeated. As shown above, the deposition of 10 nm is sufficient to entirely switch the electrode behavior to the kinetics determined by the surface layer. The accurate temperature of the sample was calculated from the YSZ offset resistance according to ref. 94. Comparing LSF and LSF-Pt2 reaction kinetics again reveals a factor of 2.5 faster reaction kinetics of the Pt surface doped material. For data analysis, the logarithm of the surface ASR was plotted *versus* the inverse temperature (see the Arrhenius diagram in Fig. 8B). The obtained plot reveals excellent linear behaviour over the whole investigated temperature range indicating an Arrhenius-type temperature dependence. The activation energy was calculated from the slope of the linear fit. For LSF an activation energy of 1.00 eV was found and LSF-Pt2 showed virtually the same activation energy of 1.03 eV. In an earlier study, the activation energy of La_0.6_Sr_0.4_CoO_3−*δ*_ was also measured in the *i*-PLD setup under the same conditions. While LSC electrodes showed even better oxygen reduction kinetics than LSF-Pt2, also an identical activation energy of 1.03 eV was found.^79^ This almost identical activation energy for all three materials, in addition to the identical *p*(O_2_) dependence mentioned above, indicates that the reaction mechanism is the same on all these perovskite-type oxygen reduction electro-catalysts. Moreover, studies on STF and LSC revealed activation energies to strongly depend on oxygen partial pressure. While usually, in accordance with this study, an activation energy of 1 eV is reported at a lower oxygen partial pressure (0.04 mbar), an activation energy of 1.6 eV for LSC^79^ and values between 1.6 eV and 2.5 eV (depending on the Fe : Ti ratio) for STF^33^ are found at ambient air. According to the literature,^33,79^ a change of the oxygen exchange reaction mechanism on the electrode surface at higher *p*O_2_ may explain the change of the activation energies. This might also be in accordance with Fig. 8A, where a change of the *p*O_2_ dependence of the ASR was found at higher *p*O_2_. Possible mechanisms and rate determining steps on LSF, LSF-Pt2 and LSC are discussed below.

## 4 Discussion

In the following, we conclude our findings regarding the superior properties of Pt doped LSF in comparison to pure LSF, suggest mechanistic explanations and connect them with recent findings in the literature. Overall, the following experimental facts have to be considered by a mechanistic explanation:

(i) Pt is incorporated into the LSF lattice in an oxidized state and no evidence for metallic Pt on the electrode surface was found (please see Fig. 4 and 5). The preferred Pt^4+^ oxidation state indicates occupation on the B-site.

(ii) The polarisation resistance of Pt surface doped LSF electrodes is about a factor of 2.5 lower than that of LSF, despite the small concentration of platinum used (please see, for instance, Fig. 7).

(iii) A few nanometers of Pt doped LSF deposited on top of undoped LSF are sufficient to achieve this excellent oxygen exchange kinetics (please see Fig. 7 and 6).

(iv)On LSF and LSF-Pt2 (and LSC in an earlier study^79^), the same activation energy of 1.00–1.03 eV of the oxygen exchange reaction was found (please see Fig. 8 B).

(v) LSF and Pt doped LSF exhibit the same partial pressure dependence of the polarisation resistance (please see Fig. 8 A).

Based on the above mentioned experimental observations, we need to find an explanation considering the significantly improved surface kinetics in the presence of platinum, while the reaction mechanism and rate limiting step remain the same, as suggested by the identical *p*(O_2_) dependence and activation energy. Especially the latter is a clear indication that in the present case platinum does not act as a classical catalyst, which would be evident by establishing a novel reaction pathway with a different activation energy and probably a different *p*(O_2_) dependence. This is in strong contrast to an earlier study where we deposited Pt nanoparticles on LSF.^69^ This caused not only a dramatic drop of the polarisation resistance but also a complete change of the *p*(O_2_) dependence (even including a change of the sign).

In the literature,^98^ the reaction rate of oxygen exchange was defolded into its different contributions and the following equation was suggested to describe the kinetics:3
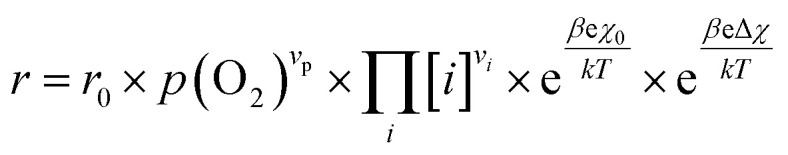


This equation includes the oxygen partial pressure *p*(O_2_), the concentrations [*i*] of the reacting species *i* (*i.e.* Fe^3+^ and oxygen vacancies for oxygen reduction), the effect of any surface potential *χ*_0_ or its change upon voltage Δ*χ* (which can become relevant if charge is transferred across the surface) and a prefactor *r*_0_ which includes kinetic contributions (chemical activation barriers) but also thermodynamic contributions due to mass action equilibria before the rate limiting step, *k* and *T* denote Boltzmann's constant and temperature. The relevant species of oxygen reduction are oxygen gas molecules or oxygen adsorbates coupled to the gas *via* a fast adsorption step, and defects at the LSF surface. The oxygen partial pressure thus enters the equation with a reaction order *n*(*ν*_*p*_), which considers the fast adsorption kinetics (if relevant), *e.g.* without site restriction we get *ν*_*p*_ = 1 for molecular adsorption. Relevant defects for oxygen reduction are particularly oxygen vacancies and Fe^3+^ ions, the latter providing the electron for reduction (thus forming Fe^4+^ = holes, which then enter the rate equation of the oxygen reduction reaction). Symbol *v*_*i*_ is the reaction order of the defect *i*, which depends on the specific mechanism and rate limiting step.

This general approach is very helpful for deducing any mechanistic conclusion from the given experimental data. It is far beyond the scope of this paper to discuss details of the specific mechanism and to identify the rate limiting step for these materials. However, the virtually identical activation energy for LSF with and without Pt as well as the same *p*(O_2_) dependence still enable important conclusions about the oxygen exchange mechanism.

Eqn (3) shows that the oxygen partial pressure enters the reaction rate twice: first it enters directly *via* the gas or adsorbate species (
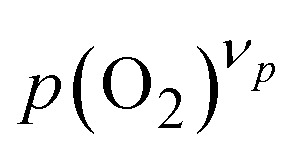
 term) and second it enters the rate indirectly *via* the partial pressure dependent defect concentrations. The latter are often rather well-known in the bulk (Brouwer diagrams, *e.g.* ref. 82 for LSF thin films), but one should keep in mind that absolute concentrations at the surface may differ from the bulk (surfaces are often more reduced). Moreover, surface segregates and additional reactions with gas contaminants may change the surface drastically and bulk defect chemistry is no longer an optimal tool for estimating the surface defect concentrations (effects of the oxygen partial pressure on the surface potential are also possible but neglected here, in accordance with ref. 21 and 86). With this in mind, we can now suggest that the identical *p*(O_2_) dependence of the two sample types, together with the same activation energy, indicates that Pt is not directly involved as a classical catalyst but changes a relevant defect concentration. Electron-delivering Fe^3+^ ions are most probably still available in high concentrations at these partial pressures and can hardly be increased substantially by Pt doping. We thus believe that the Pt doping rather increases the surface oxygen vacancy concentration. Depending on the reaction order with respect to vacancies, this might be an increase by a factor of 2.5 for *n*(*v*_*V*_) = 1 or 1.6 for *n*(*v*_*V*_) = 2. However, also some small effects of Pt^4+^ on mass action equilibria of reaction steps before the rate limiting step may be present, which would induce changes of the prefactor *r*_0_. The exact mechanism how Pt increases the surface vacancy concentration is still unknown, but it seems plausible that the incorporation of larger Pt^4+^ ions on the Fe^3+^ lattice site leads to a slight enlargement of the unit cell, which decreases the formation enthalpy of oxygen vacancies.^42,99,100^ This is also in accordance with XRD measurement, where an increase of the unit cell of Pt doped LSF compared to pure LSF was observed.

The slope in the partial pressure dependence is then largely an interplay between increasing oxygen gas concentration and decreasing vacancy concentration for increasing *p*(O_2_). The higher *p*(O_2_), the stronger decreases the vacancy concentration until it reaches a −1/2 slope in the Brouwer diagram, thus explaining the weakened slope of the reaction rate at higher oxygen partial pressures. A more detailed quantitative analysis in terms of mechanisms needs much further data (including bias dependent measurements) and is beyond the scope of this study.

However, our results reveal that Pt shows the tendency to leave the perovskite lattice of Pt doped LSF forming Pt particles on the surface, see also to the ESI,† Chapter 1. Thus, the beneficial effect of platinum (formation of oxygen vacancies) is lost and an increase of the polarisation resistance is found, which is in good accordance with our mechanistic model. In our study, Pt was chosen as a dopant as prior studies on this material combination were available,^75–77,93^ nevertheless the effect should not be limited to platinum as also other – cheaper and more stable – dopants might lead to a similar enlargement of the LSF unit cell.

## 5 Conclusions

In this study, we show how small amounts of platinum doping (0.9 at% and 1.5 at%) can significantly improve the oxygen reduction kinetics of LSF electrodes. Interestingly, Pt was not present in its otherwise typical catalytically active form as a metal, but rather it was found to be incorporated into the LSF perovskite in the oxidized form (Pt^4+^). For characterization of the electro-catalytic activity of pure and Pt doped LSF electrodes, impedance measurements were conducted *in situ* inside the chamber of the PLD, which guarantees electrodes with very clean surfaces unaltered by external degradation. Moreover, multi-layer growth of different electrode materials on the same substrate under the measurement conditions allows excellent comparability of resistance values. Despite the small amount of platinum doping in a very thin layer (5 nm of LSF-Pt2) (80 ng cm^−2^ Pt loading) about a factor of 2.5 lower polarisation resistance was measured. This significant increase in electrochemical performance was especially interesting as both pure LSF and Pt doped LSF showed the same oxygen partial pressure dependence and activation energy, which indicates that still the same mechanism and rate limiting step are active on both materials. Consequently, the addition of Pt is interpreted to increase the number of defects relevant in the reaction, more specifically the concentration of oxygen vacancies at the surface.

## Author contributions

C. R. prepared the samples, performed and analyzed the impedance measurements, proposed the initial idea for the measurements and wrote the manuscript, M. S. assisted with the *i*-PLD measurements and performed the X-ray diffraction measurements, A. N. performed and analyzed the XPS measurements, G. F. conducted the AFM measurements, M. W. conducted the ICP-MS measurements, A. L. supervised the ICP-MS measurements and was involved in the interpretation of the data, J. B. performed and analyzed the TEM measurements, C. Ra. Supervised the XPS measurements and was involved in the interpretation of the results, M. K, A. K. O., and J. F. supervised all measurements, acquired the funding and were involved in the interpretation of the results as well as the preparation of the manuscript. All authors read the manuscript.

## Conflicts of interest

There are no conflicts to declare.

## Supplementary Material

TA-010-D1TA08634K-s001
