# Supplementary material for: Performance modulation through selective, homogenous surface doping of lanthanum strontium ferrite electrodes revealed by in situ PLD impedance measurements
Source: J Mater Chem A Mater. 2021 Dec 3;10(6):2973–86. doi: 10.1039/d1ta08634k (PMC8823903; doi:10.1039/d1ta08634k)
Supplement: TA-010-D1TA08634K-s001 [file TA-010-D1TA08634K-s001.pdf]

Electronic Supplementary Material (ESI) for Journal of Materials Chemistry A.
This journal is © The Royal Society of Chemistry 2021

Supporting Information: Performance modulation through selective, homogenous surface doping of lanthanum strontium ferrite electrodes revealed by *in-situ* PLD impedance measurements

Christoph Riedl[a], Matthäus Siebenhofer [a,d], Andreas Nenning[a], Gernot Friedbacher[a], Maximilian Weiss[a], Christoph Rameshan[b], Johannes Bernardi[c], Andreas Limbeck[a], Markus Kubicek[a], Alexander Karl Opitz[a]*, Juergen Fleig[a]*

[a] Institute of Chemical Technologies and Analytics

TU Wien

Getreidemarkt 9-E164, 1060 Vienna, Austria

E-mail: christoph.riedl@tuwien.ac.at, alexander.opitz@tuwien.ac.at

[b] Institute of Materials Chemistry

TU Wien

Getreidemarkt 9-E165-PC, 1060 Vienna, Austria

[c] USTEM Universitäre Service-Einrichtung für Transmissions-Elektronenmikroskopie

TU Wien

Wiedner Hauptstrasse. 8-10, 1040 Wien, Austria

[d] CEST Kompetenzzentrum für elektrochemische Oberflächentechnologie GmbH

TFZ – Wiener Neustadt Viktor-Kaplan-Strasse 2

2700 Wiener Neustadt, Austria

1 Degradation behaviour

While numerous MIEC electrodes have already shown sufficiently fast oxygen reduction kinetics at pristine state, many of them suffer from poor stability of the polarisation resistance with degradation rates above an order of magnitude resistance increase within 10 hours being absolutely possible.[1,2] In this experiment, the degradation behaviour of LSF and LSF-Pt2 electrodes were compared *in-situ* directly in the setup of the *i*-PLD at 600 °C in synthetic air. As shown in Figure S1A, different degradation behaviour was found for LSF and LSF-Pt2 upon annealing. While, on the one hand, for LSF moderate oxygen reduction kinetics were observed at pristine state, the polarisation resistance increased only little over time (0.75 Ωcm^2 to 1.25 Ωcm^2 in 16 h). LSF- Pt2, on the other hand, showed superior oxygen reduction kinetics at pristine state ($< 0.2\ \Omega cm^2$), which increased to about 1.25 Ωcm^2 after 11 h of annealing at 600 °C. This degradation is still slow enough to warrant the $p(O_2)$ and temperature dependent measurements shown in the main text. AFM and XPS was carried out on pristine and degraded LSF-Pt1 electrodes to reveal changes of the electrode surface composition and morphology. AFM images shown in Figure S1B (pristine LSF-Pt1 electrode) and S1C (LSF-Pt1 electrode annealed for 16 h in synthetic air) expose very different surface morphologies. On pristine LSF-Pt1 electrodes flat surfaces (surface roughness $< \pm 1$ nm) with clearly visible grains were observed. XPS measurements revealed 0.9 % of the surface to be platinum, which excellently matches the bulk Pt concentration measured by ICP. On degraded LSF-Pt1 electrodes, however, numerous nanoparticles in the range of 50 nm were found by means of AFM on the surface of the electrode. In addition, XPS measurements showed a strong increase of the platinum fraction on the surface to 5.5 % together with a clear shift of the binding energy of the Pt signal to significantly lower values. (Complete electrode surface composition and XPS spectra can be found in the Supporting Information section 2). Thus, we expected the observed particles to be mainly metallic platinum. In literature, several recent studies have claimed Sr segregation to be one of the main reasons behind electrode degradation.[3–9]. Moreover, sulphur contamination[10–13] or silicon poising[14,15] may deactivate the catalytically active sides on the surface of the MIEC. In the case of LSF-Pt2 electrodes, we expect that degradation is mainly triggered by agglomeration of platinum on the surface of the electrode, especially because XPS did not show a significant increase of the Sr concentration on degraded LSF-Pt1 samples and the degradation is much more pronounced than on LSF. Moreover, in an earlier study on Pd doped

perovskite catalysts noble metal particles were found on the surface of the electrode after annealing for several hours at high temperatures in air.[16] Hence, essentially the oxygen vacancy concentration increase triggered by Pt^{4+} in the oxide seems to be largely lost when Pt agglomerates at the surface. The degradation of LSF was much less pronounced and was most probably driven by Sr segregation or poising of the electrode surface, but was not further investigated in this study.

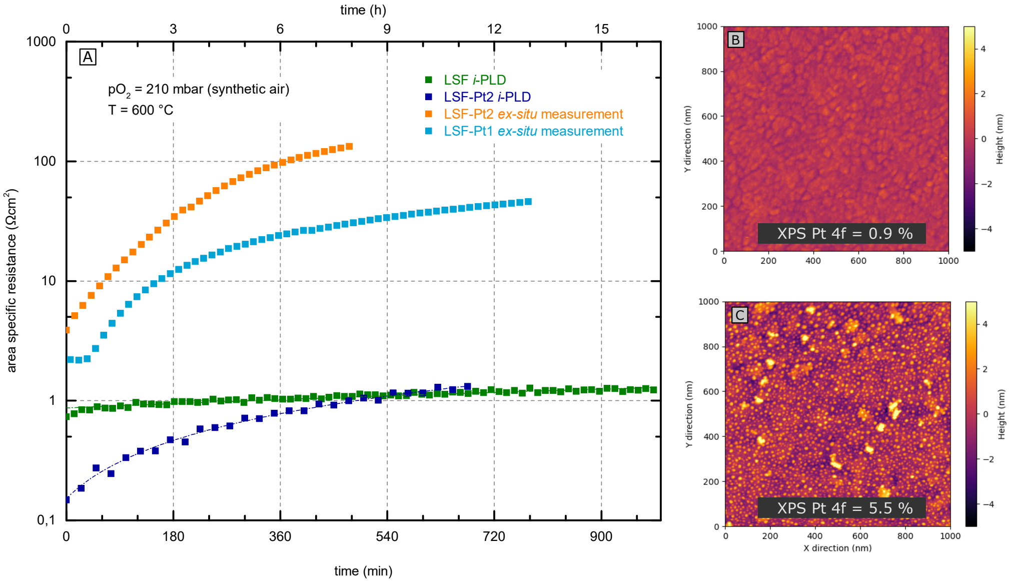

Figure S1: A) Degradation behaviour of LSF and LSF-Pt2 during *i*-PLD measurements at 600 °C in synthetic air. B) AFM image of a pristine LSF-Pt1 electrode directly after PLD deposition C) AFM image of a LSF-Pt1 electrode annealed for 16 h in synthetic air at 600 °C. The inset in figure B), C) shows the observed surface composition with platinum measured with XPS.

2 Spectra and results of *ex-situ* XPS measurements

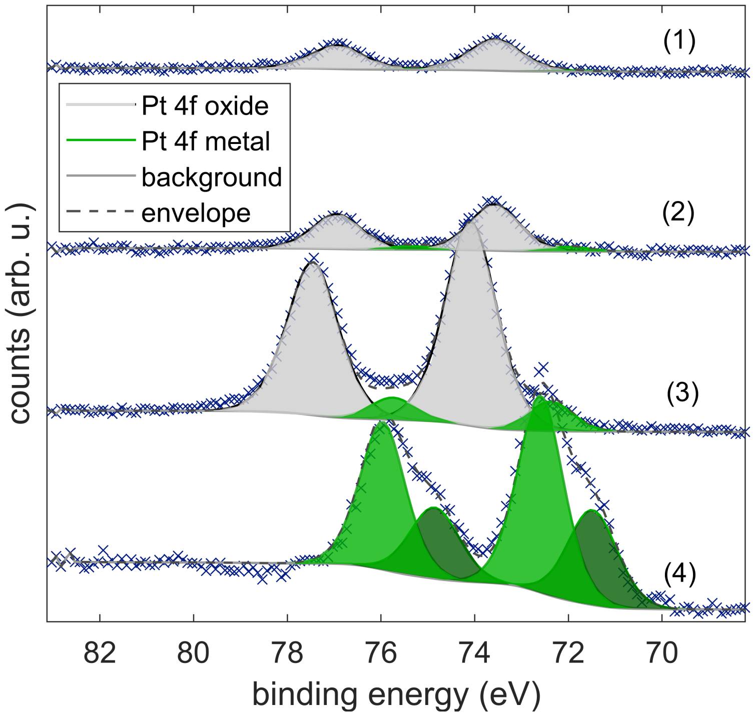

Figure S2: *ex-situ* XPS measurements (1) pristine LSF-Pt1 (2) pristine LSF-Pt1 heated up to 450 °C in UHV chamber directly before XPS measurements (3) LSF-Pt1 annealed for 16 h in synthetic air, (4) LSF-Pt1 annealed for 16 h in synthetic air, heated up to 450 °C in UHV directly before XPS measurements.

XPS was measured on pristine LSF-Pt1 electrodes (1) and on LSF-Pt1 electrodes annealed in synthetic air for 16 h (3). In addition, the same electrodes were heated up to 450 °C (heater thermocouple located close the sample) in UHV before the XPS measurements to reduce the oxidation state of platinum particles on the surface of the electrode (2) & (4). Platinum incorporated into the pervoskite host lattice is not reduced upon this conditions.

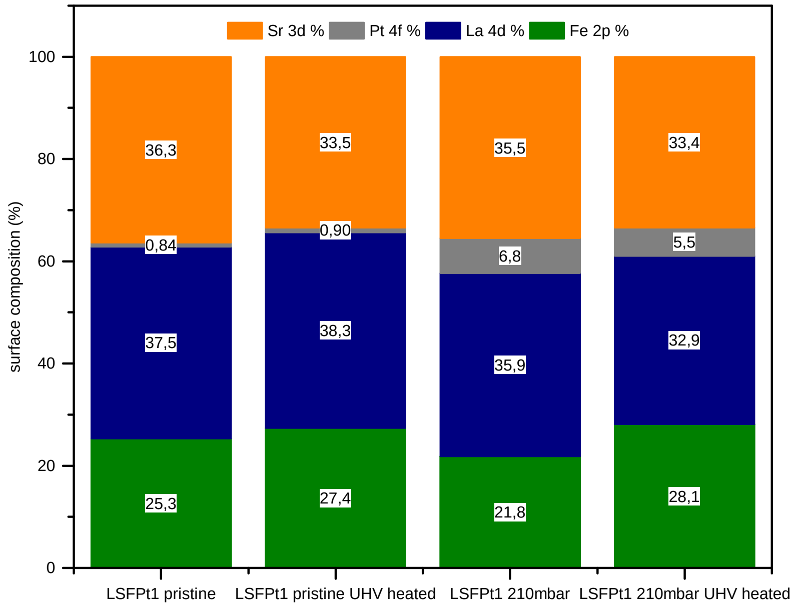

Figure S3: Calculated surface composition from *Ex-situ* XPS measurements on (1) pristine LSF-Pt1 (2) pristine LSF-Pt1 heated up to 450 °C in UHV chamber directly before XPS measurements (3) LSF-Pt1 annealed for 16 h in synthetic air, (4) LSF-Pt1 annealed for 16 h in synthetic air, heated up to 450 °C in UHV directly before XPS measurements.

3 SEM and AFM images of pristine and degraded LSF-Pt1 electrodes

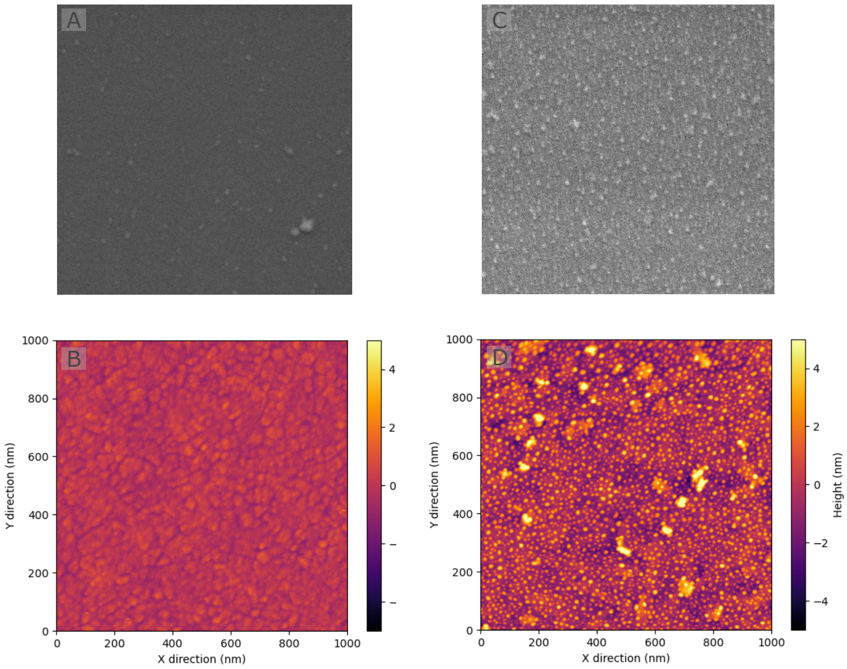

Figure S4: A) SEM image of a pristine LSF-Pt1 electrode directly after deposition B) SEM image of a LSF-Pt1 electrode, which was annealed for 16 h at 600 °C in synthetic air. C) AFM image of a pristine LSF-Pt1 electode directly after deposition D) AFM image of a LSF-Pt1 electrode,which was annealed for 16 h in synthetic air.

4 Results of AP-XPS measurements

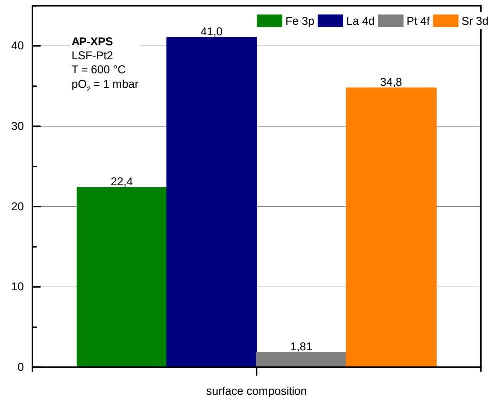

Figure S5: Surface composition of a LSF-Pt2 electrode measured at 600 °C and 1 mbar O_2

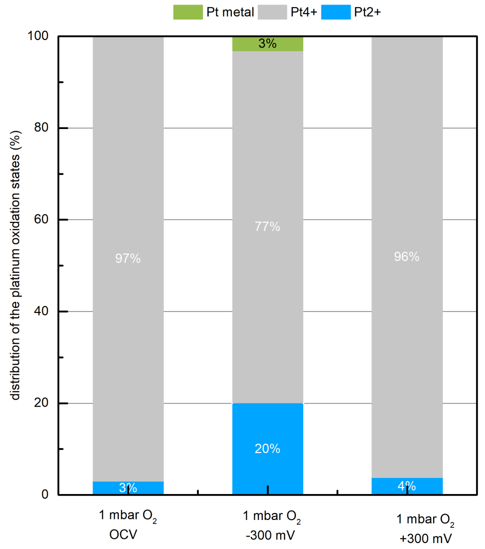

Figure S6: Distribution of the platinum oxidation states at OCV and upon application of -300 mV and +300 mV of overpotential.

5 Platinum current collector grid

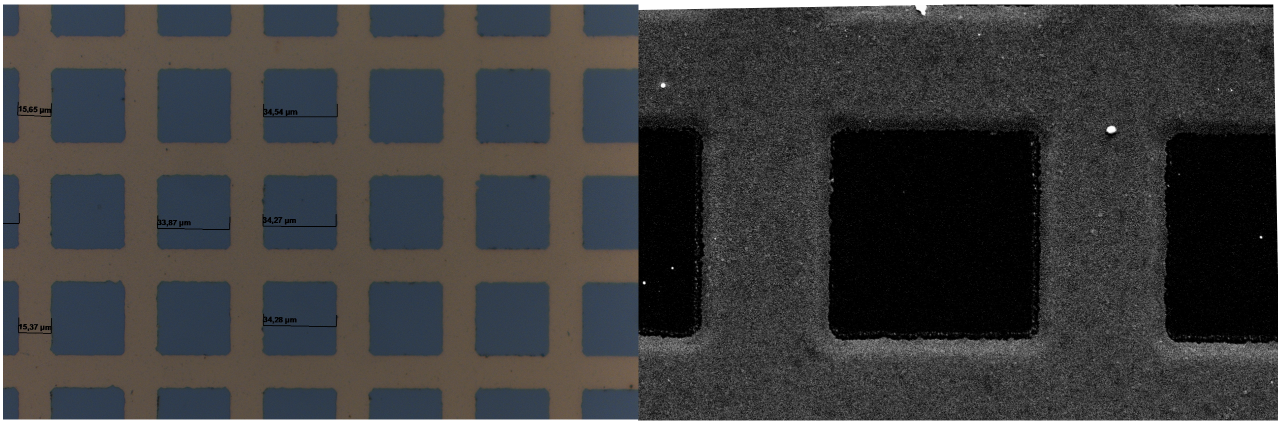

Figure S7: Platinum grid used for electrode contacting. Images from reflected light and secondary electron microscope.

References

(1) Z. Cai, Y. Kuru, J. W. Han, Y. Chen and B. Yildiz, *Journal of the American Chemical Society*, 2011, **133**, 17696–17704.

(2) G. M. Rupp, A. Limbeck, M. Kubicek, A. Penn, M. Stöger-Pollach, G. Friedbacher and J. Fleig, *J. Mater. Chem. A*, 2014, **2**, 7099–7108.

(3) M. Niania, R. Podor, T. B. Britton, C. Li, S. J. Cooper, N. Svetkov, S. Skinner and J. Kilner, *Journal of Materials Chemistry A*, 2018, **6**, 14120–14135.

(4) M. A. R. Niania, A. K. Rossall, J. A. V. den Berg and J. A. Kilner, *Journal of Materials Chemistry A*, 2020, DOI: `10.1039/d0ta06058e`.

(5) J. Druce, T. Ishihara and J. Kilner, *Solid State Ionics*, 2014, **262**, 893–896.

(6) H. Wang, K. J. Yakal-Kremski, T. Yeh, G. M. Rupp, A. Limbeck, J. Fleig and S. A. Barnett, *Journal of The Electrochemical Society*, 2016, **163**, F581–F585.

(7) M. Kubicek, A. Limbeck, T. Froomling, H. Hutter and J. Fleig, *Journal of The Electrochemical Society*, 2011, **158**, B727.

(8) M. Kubicek, G. M. Rupp, S. Huber, A. Penn, A. K. Opitz, J. Bernardi, M. Stöger-Pollach, H. Hutter and J. Fleig, *Physical Chemistry Chemical Physics*, 2014, **16**, 2715.

(9) F. Pişkin, R. Bliem and B. Yildiz, *Journal of Materials Chemistry A*, 2018, **6**, 14136–14145.

(10) A. Schmid, A. Nenning, A. K. Opitz, M. Kubicek and J. Fleig, *Journal of the Electrochemical Society*, 2020, DOI: `10.1149/1945-7111/abac2b`.

(11) E. Bucher, C. Gspan, F. Hofer and W. Sitte, *Solid State Ionics*, 2013, **238**, 15–23.

(12) C. Berger, E. Bucher, C. Gspan, A. Menzel and W. Sitte, *Journal of The Electrochemical Society*, 2017, **164**, F3008–F3018.

(13) C. Berger, E. Bucher, C. Gspan, A. Menzel and W. Sitte, *Solid State Ionics*, 2018, **326**, 82–89.

(14) M. Perz, E. Bucher, C. Gspan, J. Waldhäusl, F. Hofer and W. Sitte, *Solid State Ionics*, 2016, **288**, 22–27.

(15) E. Bucher, C. Gspan, T. Höschen, F. Hofer and W. Sitte, *Solid State Ionics*, 2017, **299**, 26–31.

(16) H. Tanaka, I. Tan, M. Uenishi, M. Kimura and K. Dohmae, *Topics in Catalysis*, 2001, **16/17**, 63–70.
